# A systematic review of specialist inpatient dementia care services versus standard inpatient dementia care in acute hospitals

**DOI:** 10.1007/s40520-018-1021-y

**Published:** 2018-09-26

**Authors:** B. M. S. McCausland, H. P. Patel, J. Amin, D. S. Baldwin, K. Loughran, V. C. Osman-Hicks

**Affiliations:** 1 University of Southampton Faculty of Medicine (Clinical and Experimental Sciences), University Hospital Southampton NHS Foundation Trust, Southampton, UK; 2 Department of Psychological Medicine, University Hospital Southampton NHS Foundation Trust, Southampton, UK; 3 Academic Geriatric Medicine, University of Southampton, University Hospital Southampton NHS Foundation Trust, Southampton, UK; 4 Medicine for Older People, University Hospital Southampton NHS Foundation Trust, Southampton, UK; 5 Medical Research Council Lifecourse Epidemiology Unit, University Hospital Southampton NHS Foundation Trust, Southampton, UK; 6 National Institute for Health Research Southampton Biomedical Research Centre, University Hospital Southampton NHS Foundation Trust, Southampton, UK

**Keywords:** Dementia, Acute hospital, Specialist dementia ward

## Abstract

**Background:**

Specialist inpatient dementia units (SIDU) have been developed to address adverse outcomes often experienced by people living with dementia admitted to acute hospitals. However, the evidence base of their effectiveness remains limited.

**Aim:**

To review the current literature to establish the comparative effectiveness of acute hospital SIDU vs. standard ward care (SWC).

**Methods:**

We did an online search of 12 biomedical databases from inception to 31st October 2017. Studies of inpatients with any form of dementia in acute hospitals, published in English language peer-reviewed journals, using experimental, observational or qualitative study designs, comparing SIDU with SWC and which measured any qualitative or quantitative outcome of the patient or carer experience were included in the search criteria. We used a standardised data extraction and appraisal form.

**Results:**

Three of 46 full-text studies evaluated were suitable for analysis. Due to study heterogeneity, pooled odds ratios were only possible for mortality [OR 1.06 (CI 1.0–1.4)]. Otherwise, a narrative synthesis was performed. Although quantitative measures of length of stay, mortality and behavioural and psychiatric symptoms of dementia are not significantly lower, SIDU are associated with greater patient and carer satisfaction, reduced readmission rates, more accurate and comprehensive assessment processes, documentation of resuscitation decisions, and increased rates of discharge to the patient’s own home.

**Conclusions:**

Although SIDU may be associated with improved care outcomes, the current evidence of their effectiveness is markedly limited. Further research and service evaluation of SIDU as a method for providing high-quality dementia care in acute NHS Trusts is needed. PROSPERO: CRD42017078364.

## Introduction

Dementia represents a significant and increasing health and social care problem in the context of an ageing population [1, 2]. Approximately 850,000 people in the UK live with dementia, costing the UK economy an estimated £26 billion annually [3, 4]. Recent data indicate that 86.7% of patients aged over 75 admitted to UK NHS Trusts for longer than 72 h were identified as potentially having dementia [5]. Acute hospital admission for patients living with dementia is associated with adverse outcomes from increased length of stay (LOS), morbidity and mortality [6]. The continued assessment and improvement of NHS dementia care is therefore necessary [7].

Multidisciplinary, specialist inpatient dementia units (SIDU) have been developed within acute Trusts for patients with dementia and concomitant acute medical illness, whose needs are more complex. Their aim is to increase patient dignity and autonomy with person-centred care [8] provided by staff from both psychiatric and geriatric care backgrounds, trained in managing the behavioural and psychological symptoms of dementia (BPSD) and delirium [9]. These symptoms are often difficult to identify and manage for untrained staff, particularly within the pressured environment of acute hospitals [10, 11]. If SIDU can reduce LOS by 1 week per patient, the NHS could save up to £80 million yearly [12].

The aim of this systematic review was to determine whether acute hospital SIDU are effective when compared with standard inpatient ward care (SWC) in improving outcomes for patients living with dementia.

## Methods

### Search strategy and selection criteria

We attempted to locate all peer-reviewed published studies meeting the selection criteria: (1) included men and/or women of any age with any form of dementia, (2) presented the results of peer-reviewed English language research using the following study designs: experimental studies (e.g., randomised controlled trials, non-randomised controlled trials, parallel group studies), before and after studies, interrupted time series studies, case note reviews, cohort studies, case–control studies, cross-sectional studies, case studies, case series, or any qualitative design (e.g., in-depth interviews, focus groups); (3) included participants who were inpatients of an acute hospital; (4) compared SIDU with SWC; (5) measured qualitative or quantitative outcome measures of patient and/or carer experience of the hospital stay. PRISMA reporting guidelines were followed [13, 14]. PROSPERO registration: CRD42017078364.

General discussion papers, comments, letters, book chapters, single case studies, national reports and published conference abstracts were excluded. As there are no gold diagnostic standards aside from post mortem examination, searches were not restricted to studies that used a validated dementia diagnostic method. If stated, the method of assessing dementia was recorded. As we were focusing on acute Trusts in the UK and Ireland, we did not include non-English language studies. If multiple eligible publications from the same study were identified, the one with the largest sample size was included to minimise duplication.

The search strategy comprised (1) electronic searches of 12 biomedical databases (Cochrane, Medline, Embase, Web of Science, Psychinfo, Health Management Information Consortium, British Nursing Index, Cumulative Index to Nursing and Allied Health Literature, Science Direct, Allied and Complementary Medicine Database, Health Business Elite and PubMed), from their inception to 31st October 2017; (2) citation tracking by manual reference list screening of included studies; (3) expert recommendations (Professors Rowan Harwood and Sube Bannerjee).

### Search terms

Dementia search terms were adapted from a Cochrane systematic review [15]. These were combined with MESH subject heading terms for dementia and health care services, then limited to acute hospitals or inpatient settings, whichever yielded most results, “Appendix 1: Search terms for replication of review”.

#### Data extraction and quality appraisal

Identified abstracts were downloaded to Endnote© software (Thompson Reuters, Version X7) and assessed against the inclusion criteria. A random selection of 10% of the abstracts was screened independently as a quality check. Potentially eligible studies were downloaded and evaluated against a standardised inclusion checklist. A standardised data extraction form was then used (“Appendix 2: Checklist and data extraction form”). Excluded references were categorised by the primary reason for exclusion. If necessary, the corresponding authors were contacted for clarification or raw data.

Two reviewers independently methodologically assessed the included studies using a standardised appraisal form with a maximum score of 40, developed by Trevillion et al. using criteria adapted from validated tools [16–18] (“Appendix 3: Quality appraisal form”). The overall study quality was reported for all included studies.

### Data analysis

Descriptive analyses were conducted to summarise the included studies. Forest plots were generated using primary data extracted from the studies using DistillerSR Forest Plot Generator from Evidence Partners. Studies that scored poorly in domains relating to bias were not included in the meta-analysis. Funnel plots for detecting publication bias, Cochrane’s *I*^2^ statistic for quantification of study heterogeneity and meta-analyses were not performed as not enough studies met the inclusion criteria.

## Results

**Fig. 1:**
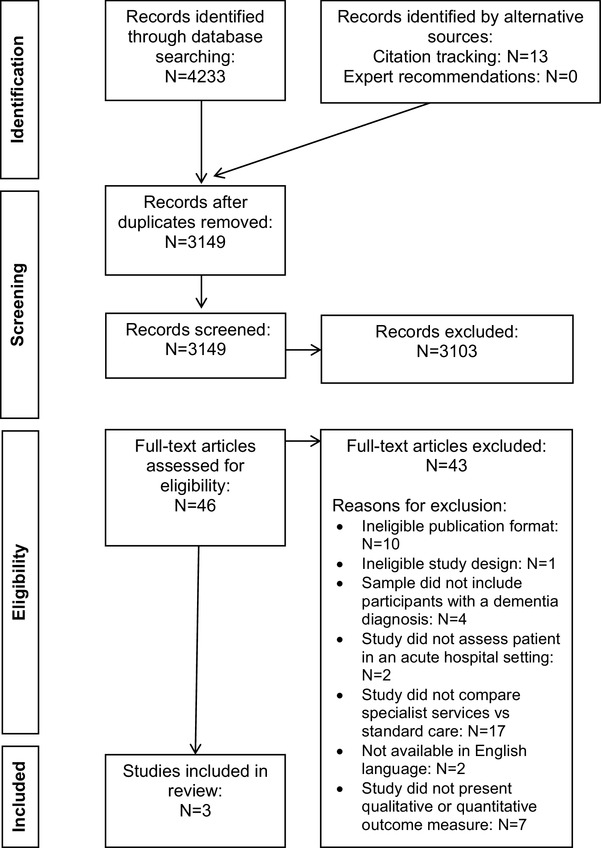
Flow diagram of literature search, including the results of the study selection strategy, numbers screened and excluded at each stage and reasons for full-text article exclusion

The results of the study selection strategy and reasons for exclusion are presented in Fig. 1. Only three studies qualified for inclusion, with little consistency in their outcome measures [19–21]. This heterogeneity meant that aside from mortality, the data were not suitable to pool for meta-analysis. A narrative synthesis of the remaining data was performed. The study characteristics are summarised in Table 1. Simplified schematic results for comparison are given in Table 2; excluding the study by Spencer et al. [19] as their qualitative results could not be similarly summarised. The combined result for the critical appraisal is included in Table 1. None of the included studies were excluded for scoring poorly on quality.

**Table 1:** Summary table of included articles

Author (year)	Where was the study done	Clinical setting and PICO	Sampling method and follow-up period	Dementia assessment method	Sample size, age (years) and gender of participants	Which patient outcomes were measured	Quality appraisal score
Briggs et al. [20]	Ireland and Northern Ireland	All acute public hospitals in Ireland and Northern Ireland PICO: in patients with dementia, does admission to a specialist geriatric medicine ward improve quantitative acute care outcomes vs. SWC?	Systematic: national audit data Follow-up period: 1 year (2013–2014)	Not specified	*N* = 900 total, *N* = 150 on specialist geriatric ward (17%), *N* = 750 (83%) on general medical/surgical wards Mean age 83.0 (SD 7.1) for all participants Not disaggregated by gender	Quantitative outcomes Admission length Mortality Behavioural and psychiatric symptoms Incidence of delirium during admission Psychotropic medications Accurate recording of medications, co-morbidities and collateral history Documented decision about resuscitation status Compliance with multidisciplinary assessments Qualitative outcomes Not included	78% (31/40)
Goldberg et al. [21]	UK	Single acute General Hospital (not named) PICO: in patients with cognitive impairment, does care on a specialist medical and mental health unit in a general hospital give better qualitative and quantitative outcomes vs. SWC?	Convenience sampling Follow-up period: 1 year (2010–2011)	Identified by clinicians as “confused” on admission—encompassing both delirium and dementia	*N* = 874 approached, *N* = 600 agreed to participate *N* = 310 on specialist unit, *N* = 290 on standard care wards (acute geriatric/general medical) Median age 85 in both groups [inter-quartile range (IQR) 80–88 on specialist unit, IQR 80–89 on standard care wards] Males *N* = 288, females *N* = 312	Quantitative outcomes Number of days spent at home in 90 days after randomisation (encompassing death, time in hospital, readmissions, rehabilitation and new care home placement) Behavioural and psychological symptoms Falls Physical disability Cognitive impairment Qualitative outcomes Patient quality of life Carer strain index Carer psychological well-being Carers’ satisfaction with care Patients’ mood and engagement on the wards (direct observation of a random subsample of patients)	85% (34/40)
Spencer et al. [19] N.B. this is the qualitative arm of the Goldberg et al. study above	UK	Single acute General Hospital (not named) PICO: in carers for patients with delirium or dementia, does care on a specialist medical and mental health unit in a general hospital result in better reported qualitative outcomes vs. SWC?	Convenience sampling Follow-up period: 1 year (2010–2011)	Not specified	*N* = 40 patients and *N* = 40 carers included Mean age 87 on specialist unit (range 83–97) vs. 85 (range 69–95) on standard care wards Males *N* = 18, females *N* = 22 (specialist unit: males *N* = 7, females = 13; standard care males = 11, females = 9)	Quantitative outcomes Not included Qualitative outcomes Face-to-face semi-structured interviews with carers pooled into six themes 1. Activities and boredom 2. Staff knowledge 3. Dementia, dignity and fundamental care 4. Ward environment 5. Communication between carers and staff 6. Carer expectations	70% (28/40)

The information is presented here as it is given in the included articles, meaning there are some differences in comparison data, e.g., median vs. mean ages. PICO: patient, intervention, comparison and outcome; we have summarised the PICO questions for each paper for clarity and as part of the critical appraisal process

**Table 2:** Schematic results summary

Outcome	Briggs et al. [20]		Goldberg et al. [21]	
	SIDU	SWC	SIDU	SWC
**Quantitative**				
Length of stay	↔	↔	↔	↔
Days spent at home	–	–	↔	↔
Discharged to their own home	–	–	↑	↓
Discharged to new care home	–	–	↓	↑
Rate of readmission	–	–	↓	↑
Mortality	↔	↔	↔	↔
Rates of BPSD	↔	↔	↔	↔
Incidence of delirium^a^	↑	↓	–	–
New antipsychotic medications^a^	↑	↓	–	–
Overall antipsychotic prescription rates	↔	↔	–	–
Documentation of treatment decisions	↔	↔	–	–
Accurate drug history^a^	↑	↓	–	–
Accurate co-morbidities documented^a^	↑	↓	–	–
Collateral history taken regarding cognition^a^	↑	↓	–	–
Single plan for discharge^a^	↑	↓	–	–
Resuscitation status documented^a^	↑	↓	–	–
Barthel index score	–	–	↔	↔
MMSE score	–	–	↔	↔
**Qualitative**				
Patient quality of Life	–	–	↔	↔
Carer strain index	–	–	↔	↔
Carer psychological well-being	–	–	↔	↔
Patient positive mood/engaged*	–	–	↑	↓
Patient active	–	–	↑	↓
Patient interacting with others socially	–	–	↑	↓

SIDU: Specialist Inpatient Dementia Units (as defined by the source papers). The symbols ↑ for more, ↓ for less and ↔ for equivalent outcomes are used to summarise the results simply. A dash (–) is used to denote that this was not measured by the study

*SWC* standard ward care

*P value < 0.05

^a^Odds ratio > 1

### Results synthesis

**Fig. 2:**
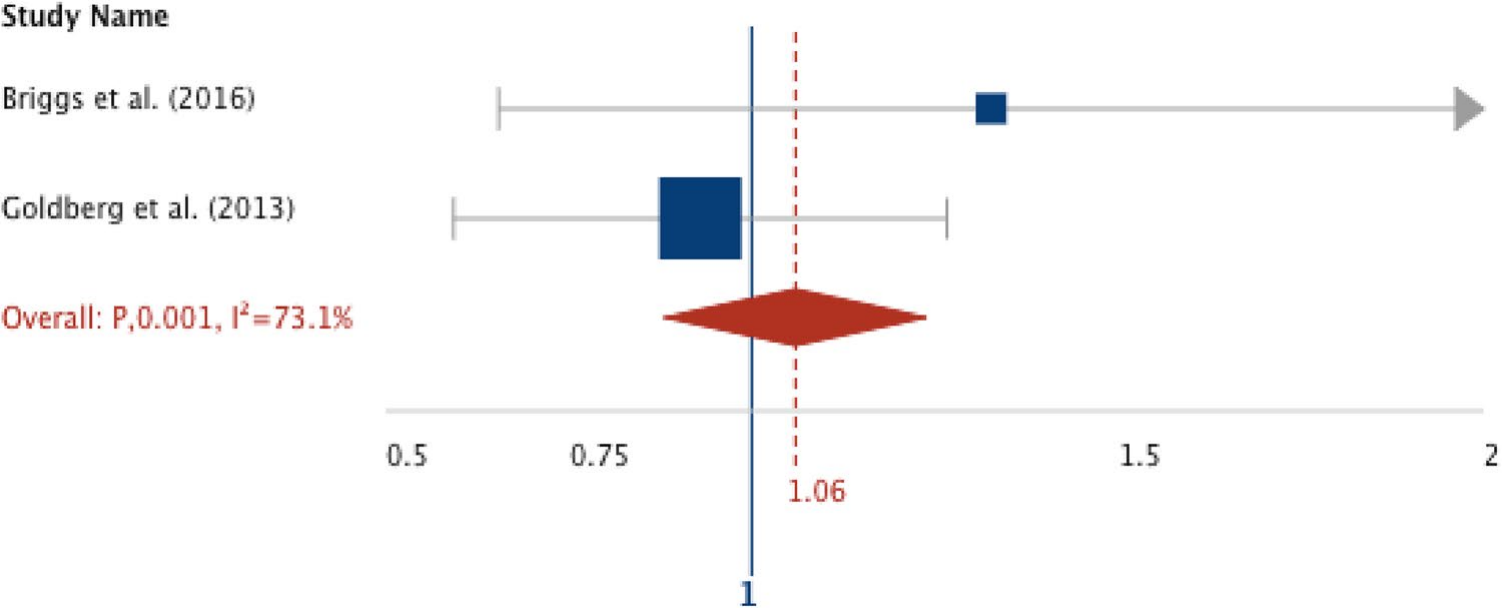
Forest plot odds estimates for mortality comparing SIDU with SWC. No significant difference was found by either in mortality between the SIDU and SWC; Briggs et al. [20] (SIDU 9% vs. SWC 8%, OR 1.21; CI 0.65–2.22; *P* = 0.55); Goldberg et al. [21] (22% SIDU vs. 25% for SWC; OR 0.87; CI 0.60–1.23; *P* = 0.46). The pooled odds ratio for mortality was 1.06 (CI 1.0–1.4)

Only LOS, rates of BPSD and mortality were measured by more than one study. From these, only mortality data allowed the generation of odds ratios and a Forest plot, Fig. 2. No significant difference was found in mortality between the SIDU and SWC in either study; Briggs et al. [20] (SIDU 9% vs. SWC 8%, OR 1.21; CI 0.65–2.22; *P* = 0.55); Goldberg et al. [21] (22% SIDU vs. 25% for SWC; OR 0.87; CI 0.60–1.23; *P* = 0.46). The pooled odds ratio was 1.06 (CI 1.0–1.4).

Neither quantitative study found a significant difference in LOS between the SIDU and SWC (SIDU 28.5 ± 31.4 days vs. SWC 25.1 ± 38.7, *P* = 0.471) [22]; (SIDU 16 vs. SWC 16 median days; adjusted CI 0.93–1.23, *P* = 0.32) [21]. However, Briggs et al. [20] found that fewer admissions on the SIDU lasted less than 7 days (SIDU 22.0% vs. SWC 28.3%, *P* = 0.250) and Goldberg et al. [21] found that the SIDU had a non-significantly lower rate of readmission (32 vs. 35% for SWC; CI − 10 to 5%). Neither study found that rates of BPSD varied significantly between SIDU and SWC; SIDU 30% vs. SWC 24%, (OR 1.36; CI 0.88–2.10) [22]; SIDU 18.5 vs. SWC 17, median number of recorded symptoms at 90 days (CI − 5 to 7.5, *P* = 0.77) [21].

### Quantitative outcomes

Briggs et al. [20] found the incidence of delirium was slightly greater on SIDU (SIDU 46 vs. 33%, OR 1.70; CI 1.14–2.53), as was the rate of new prescriptions of antipsychotic medications (SIDU 50% vs. SWC 34%, OR 1.95; CI 1.08–3.51). Overall antipsychotic prescription rate differences between the wards were non-significant (SIDU 37% vs. SWC 38%, OR 0.96; CI 0.66–1.38) and there was little difference between the wards in documenting the reasoning behind the prescriptions (SIDU 60% vs. SWC 59%, OR; CI 0.55–1.98). SIDU patients more often had an accurate drug history documented (SIDU 97% vs. SWC 89%, OR 3.55; CI 1.41–8.92), accurate co-morbidities listed (SIDU 81% vs. SWC 79%, OR 1.62; CI 0.98–2.68) and had a recorded collateral history regarding cognitive impairment (SIDU 38 vs. 25%, OR 1.85; CI 1.28–2.68). They were also more likely to have documented discharge plans (SIDU 66 vs. 45%, OR 2.38; CI 1.58–3.60) and resuscitation status decisions (SIDU 39% vs. SWC 34%, OR 1.23; CI 0.82–1.84).

Goldberg et al. [21] found no significant difference in days spent at home 90 days post randomisation (SIDU 51 vs. 45 days median for SWC; CI − 12 to 24, *P* = 0.3). SIDU patients were non-significantly more likely to be discharged to their original home (74 vs. 70% for SWC; CI − 3 to 11%) and less likely to go to a new care home (20 vs. 28% for SWC; CI − 16 to 0%). There were no significant differences in Barthel index scores for physical disability (mean scores: SIDU 11.6/20 vs. 11.6/20 for SWC; adjusted CI − 1.1 to 0.8, *P* = 0.78) and Mini-Mental State Examination (MMSE) for cognitive impairment (SIDU 16/30 vs. SWC 16/30 median score; CI − 4 to 2, *P* = 0.83).

### Qualitative outcomes

Goldberg et al. [21] found no significant difference at 90 days in patient’s quality of life (QOL) using multiple measures, carer strain index (SIDU 5.7/13 vs. SWC 5.8/13; adjusted CI − 0.49 to 1.04, *P* = 0.48) or carer psychological well-being [SIDU 12.5 (GHQ-12—out of total 36) vs. SWC 12 (GHQ-12/36); adjusted CI 1.0–1.23, *P* = 0.05]. More carers were happy with the care received on the SIDU (91% satisfied overall vs. 83% on SWC, CI 2–15%; *P* = 0.004). However, both study groups included care givers who were very dissatisfied with the quality of care received. The highest percentages of very unsatisfied carer responses for both were around communication and keeping carers informed (SIDU 11% vs. SWC 17% ‘very unsatisfied’) and discharge arrangements (SIDU 12% vs. SWC 19% ‘very unsatisfied’). In a subsample of patients, mood and engagement was represented by the proportion of time that a behaviour was observed during the designated period; SIDU patients were significantly more often in a positive mood/engaged (SIDU 79% vs. SWC 68%; CI 2–20, *P* = 0.03), with trends for being more active (82% SIDU vs. 74% SWC; CI − 2 to 16, *P* = 0.10) and interacting with others (47% SIDU vs. 39% SWC; CI − 3 to 19; *P* = 0.06).

Spencer et al. [19] performed a qualitative study of 40 carers’ views of their experience of the Goldberg et al. [21] SIDU. The themes from semi-structured interviews included activities and boredom, staff knowledge, dementia, dignity and fundamental care, ward environment, communication between carers and staff and carer expectations. Carers of patients on SIDU commented their relatives were more often engaged in activities, whereas the SWC carers more often stated that their relatives had little to do. Staff on the SIDU were described as patient and compassionate with good knowledge of how to care for people with dementia, particularly regarding wandering and BPSD, displaying personalised support. This was the opposite for SWC, where carers felt the staff sometimes had negative attitudes towards dementia care, ignoring or shouting at the patients; particularly if they were showing challenging behaviours. Some carers felt they had to provide their relative one-to-one care as the ward staff were inexperienced.

Both carer groups had some negative comments about dignity and privacy, including inadequate personal hygiene care and lack of privacy when ‘toileting’. Both groups were happy with the meals provided and efforts taken to offer alternatives if their relative had reduced appetite. However, neither was completely satisfied with the level of personal assistance given for eating and drinking. Both ward environments were felt to be clean, but the personalised touches on the SIDU were appreciated by the carers. Both SIDU and SWC carers wanted more communication with the ward staff; their main concern being feeling uninformed about their relatives’ care and discharge. Both groups had positive experiences of interactions with the staff. However, poor relationships with staff or certain staff members were associated with greater general dissatisfaction with the level of care provided. It was commented that despite some measures being taken to understand patients’ personal lives, particularly on the SIDU, the typically short LOS on acute wards made it difficult for staff to get to know their patients.

Overall, there was greater satisfaction with the level of care provided by the SIDU than by SWC. To address unmet expectations, carers were asked to suggest improvements. These included staff introducing themselves, increased stimulation for patients, allowing carers to attend ward rounds, extending visiting hours, using named nurses, daily updates from staff and having a separate bay for patients with more BPSD.

## Discussion

The SIDU model of care has been developed within acute Trusts as a means to improve the quality of care delivered and optimise flow through the hospital for people with dementia. However, due to the limited number of eligible studies, this review found no significant differences in rates of BPSD, mortality and LOS between SIDU or SWC from either study measuring quantitative outcomes [20, 21]. As no other measure was used consistently across the eligible studies, the results of other quality and flow outcomes are from individual studies.

This review cannot be used to draw firm conclusions about SIDU care and whether they should be established more widely. Nevertheless, it appears that more patients are being discharged to their own homes from SIDU, fewer to care home placements and that SIDU are associated with lower rates of readmission to hospital. This clearly has benefits to the acute trust as well as to the health economy. The SIDU model is associated with better recorded plans for discharge and recording of drug, medical and collateral histories and of resuscitation decisions. The higher incidence of delirium and of new antipsychotic prescriptions on SIDU found by Briggs et al. [20] may reflect more accurate recognition and treatment of delirium on SIDU compared with SWC, possibly be due to differences in staff expertise. Goldberg et al. [21] found that patients on the SIDU were more often in a positive mood, active and interacting with others than SWC patients. Overall carers were more satisfied with the care received on the SIDU, although both SIDU and SWC groups generated areas for improvement, and neither showed quantitative difference in measures of long-term patient QOL or carer strain and psychological well-being [19].

### Critical appraisal

All three original studies were limited by omitting the definition of dementia used to classify their participants. Briggs et al. [20] did not record the severity of dementia which may have confounded their results. They studied patients admitted from home rather than care homes, and used the prevalence of BPSD as a proxy measure for dementia severity, stating that as there was no significant baseline difference between groups, any confounders would be equally distributed and therefore not affect the analysis.

Briggs et al. [20] used retrospective data. This is reliant on accurate and thorough documentation of the care given throughout a patient’s admission, which is often not completed. The authors argue that this is likely to be an issue for any similarly designed study and will have affected both SIDU and SWC equally, being therefore unlikely to significantly skew their results.

Goldberg et al. [21] and Spencer et al. [19] studies are generated from the same randomised controlled trial; the former presenting quantitative and qualitative outcomes from their entire study, the latter presenting the results of a smaller, more in-depth qualitative arm. Both studies were limited by differences between the groups at baseline due to pragmatically having to recruit participants after randomisation because of pressures on acute unit beds. This was adjusted for in the analysis, but may have introduced confounders.

Following up people with dementia is difficult as they are often frail and may move frequently between their home, healthcare systems and care placements. There are also ethical concerns relating to fluctuating capacity to consent to inclusion in a prolonged trial [21, 23, 24]. Goldberg et al. [21] used statistical imputation to address their missing follow-up data, a model which replaces the missing value(s) with an estimate based on known results [25]. Although this is an established method of minimising bias introduced by missing data, it would have been preferable to have the complete data set to increase the likelihood of statistically significant results [26].

As Briggs et al. [20] used data from a multi-centre systematic audit in Northern Ireland and Ireland, it is likely that their results are externally valid. However, the other two studies are from the same single hospital in the UK and so their results may not be generalisable.

### Strengths and limitations of this review

This review expands on previous research assessing the efficacy and cost-effectiveness of SIDU. To our knowledge it is unique in being a systematic analysis and appraisal of this literature. The protocol was published on PROSPERO for transparency and replication, and PRISMA reporting guidelines were followed [13, 14]. The searches and quality appraisal were checked and performed by an independent reviewer to generate a more rigorous result. The data extraction and critical appraisal tools used are standardised and have been piloted previously, with good reliability [27]. Direct correspondence with experts ensured we had not missed unpublished, potentially eligible studies.

Publication and reporting bias may have affected our results as we did not include non-English language studies, and due to the general preferential publication of studies with positive results [28]. This review is limited by the lack of studies eligible for inclusion, meaning we are not able to infer direction of causality between SIDU and outcomes, or make definitive conclusions about the relative advantages or disadvantages of SIDU.

## Conclusion and future research

Although there is little consistent evidence that SIDU are superior to SWC, this more person-focused form of clinical care for people with dementia appears to be associated with greater patient and carer satisfaction, possible reduced readmission rates, more accurate history taking and documentation of resuscitation decisions and increased rates of discharge to the patients’ own home. Although mortality data was comparable, SIDU may represent a higher quality model of care for patients living with dementia.

Acute Trusts need to develop and demonstrate ‘gold standard’ dementia care models. Whilst quantitative measures such as LOS are important in evaluating service delivery, qualitative assessments are vital in ascertaining broader aspects of clinical care such as maintenance of dignity and autonomy.

The surprising paucity in eligible studies of SIDU directly contradicts the growing number of older people living with dementia admitted acutely. Hospitals nationwide need to develop innovative ways to provide high-quality specialist dementia care in line with NHS and Royal College standards, whilst maintaining flow and avoiding inappropriate readmissions [29]. It is vital to publish more research and service evaluation in this area.

## Relevance to key groups

These findings are relevant to any involved in developing dementia services, from healthcare workers to commissioning groups and policy makers.

Summary

- What is known already:
  - Dementia in acute NHS hospitals is a growing challenge which needs to be addressed to meet the increasing need
  - SIDU have been developed to tackle the health inequalities experienced by people with dementia during acute admissions
- What this review adds:
  - Despite limited eligible studies, we can infer that some outcomes are improved by SIDU, such as lower rates of admission to a care home, rates of readmission and of failed discharge from hospital
- What needs to be further investigated:
  - There needs to be further investigation of the efficacy and acceptability of these SIDU if they are being offered as a method nationally for improving dementia care in acute NHS Trusts
- Our future research aims:
  - We will conduct a service evaluation of our new SIDU (‘Enhanced Dementia Care Ward’) as informed by this review, evaluating dementia care by comparing the SIDU with general medicine and geriatric ward care in a busy Tertiary Care Centre in Southampton, UK.

## Appendix 1: Search terms for replication of review

This example was used on the Psychinfo database:

exp dementia/ or *"alzheimer’s disease”/ or *"cognitive impairment”/ or *"vascular dementia”/ or *"senile dementia”/ or *"dementia with lewy bodies”/ or (“dementia” or “amnestic, cognitive disorders” or “alzheimer” or “ad” or “lewy body” or dlb or lbd or ftd or ftld or “frontotemporal lobar degeneration” or “frontotemporal dementia” or “cognitive impairment” or “memory complaint, decline or disorder”).ti,ab) and (exp “health care services”/ or (exp “quality of care”/ or exp “health care services”/ or exp “health care delivery”/ or exp “health service needs”/ or exp “integrated services”/ or exp “mental health programs”/ or exp “quality of services”/))) and (acute hospital).ti,ab”.

## Appendix 3: Quality appraisal form

The critical appraisal tool for prevalence studies, developed by Loney et al. [17], incorporates a number of sources on study methodology from the Critical Appraisal Skills Programme checklists, sources on confounding and attrition and on quality rating of diagnosis ascertainment Downs, Black [16–18].

Please complete part 1 for all study designs and complete the relevant sections for part 2, specific to study design.

Score the answer to each question by ticking 0, 1 or 2:

0—study does not meet criteria/answer question

1—Study partially meets criteria/gives a partially satisfactory answer to the question

2—Study fully meets criteria/gives a fully satisfactory answer to the question

## References

[1] Parkin E, Baker C (2018) Dementia: policy, services and statistics. Briefing paper Number 07007, 10 July 2018, House of Commons Library. Available from http://researchbriefings.files.parliament.uk/documents/SN07007/SN07007.pdf (original 2016 version of this source Accessed Dec 2017, updated document Accessed Sept 2018)

[2] Department of Health (2015). Prime Minister’s challenge on dementia 2020.

[3] National Health Service England (2017) Dementia. Available from https://www.england.nhs.uk/mental-health/dementia/. Accessed Dec 2017

[4] Prince M, Knapp M, Guerchet M, McCrone P, Prina M, Comas-Herrera A, Wittenberg R, Adelaja B, Hu B, King D, Rehill A, Salimkumar D (2014). Dementia UK: Update.

[5] National Health Service England (2017) Dementia assessment and referral data collection—Q2 2017–18. Available from https://www.england.nhs.uk/statistics/2017/12/06/dementia-assessment-and-referral-data-collection-q2-2017-18/. Accessed Dec 2017

[6] Reynish EL, Hapca SM, De Souza N, Cvoro V, Donnan PT, Guthrie B (2017). Epidemiology and outcomes of people with dementia, delirium, and unspecified cognitive impairment in the general hospital: prospective cohort study of 10,014 admissions. BMC Med.

[7] O’Shea E, Manning E, Ross E, McErlean S, Timmons S (2015). Northern Ireland audit of dementia care in Acute Hospitals.

[8] Harwood R, Porock D, King N, Edwards G, Hammond S, Howe L, Russell C, Howard S, Jones R, Morrant J (2010) Development of a specialist medical and mental health unit for older people in an acute general hospital. University of Nottingham Medical Crises in Older People. Discussion paper series. ISSN 2044-4230. Available from https://www.nottingham.ac.uk/mcop/documents/papers/issue5-mcop-issn2044-4230.pdf. Accessed Dec 2017

[9] Royal College of Psychiatrists (2005) Who cares wins: improving the outcome for older people admitted to the general hospital: guidelines for the development of Liaison Mental Health Services for older people. Report of a Working Group for the Faculty of Old Age Psychiatry, Royal College of Psychiatrists. ISBN 0 85316 253 0. Available from https://www.rcpsych.ac.uk/pdf/whocareswins.pdf. Accessed Dec 2017

[10] Digby R, Lee S, Williams A (2017). The experience of people with dementia and nurses in hospital: an integrative review. J Clin Nurs.

[11] Moonga J, Likupe G (2016). A systematic literature review on nurses’ and health care support workers’ experiences of caring for people with dementia on orthopaedic wards. J Clin Nurs.

[12] Lakey L (2009) Counting the cost: caring for people with dementia on hospital wards. Alzheimer’s Society. Available from https://www.alzheimers.org.uk/sites/default/files/2018-05/Counting_the_cost_report.pdf. Accessed Dec 2017

[13] Moher D, Liberati A, Tetzlaff J, Altman DG (2009). Preferred reporting items for systematic reviews and meta-analyses: the PRISMA statement. PLoS Med.

[14] Stroup DF, Berlin JA, Morton SC, Olkin I, Williamson GD, Rennie D, Moher D, Becker BJ, Sipe TA, Thacker SB (2000). Meta-analysis of observational studies in epidemiology: a proposal for reporting. Meta-analysis of observational studies in epidemiology (MOOSE) group. JAMA.

[15] Fage BA, Seitz DP, Gill SS, Herrmann N, Smailagic N, Chan CCH, Nikolaou V (2013). Mini-Cog for the diagnosis of Alzheimer’s disease dementia and other dementias within a community setting (Protocol). Cochrane Database Syst Rev.

[16] Downs SH, Black N (1998). The feasibility of creating a checklist for the assessment of the methodological quality both of randomised and non-randomised studies of health care interventions. J Epidemiol Community Health.

[17] Loney PL, Chambers LW, Bennet KJ, Roberts JG, Stratford PW (2000). Critical appraisal of the health research literature: prevalence or incidence of a health problem. Chronic Dis Can.

[18] Saha S, Chant D, Welham J, McGrath J (2005). A systematic review of the prevalence of schizophrenia. PLoS Med.

[19] Spencer K, Foster P, Whittamore KH, Goldberg SE, Harwood RH (2013). Delivering dementia care differently—evaluating the differences and similarities between a specialist medical and mental health unit and standard acute care wards: a qualitative study of family carers’ perceptions of quality of care. BMJ Open.

[20] Briggs R, O’Neill D, Kennelly SP, O’Shea E, De Siun A, Gallagher P, Timmons S (2016). Does admission to specialist geriatric medicine wards lead to improvements in aspects of acute medical care for patients with dementia?. Int J Geriatr Psychiatry.

[21] Goldberg SE, Bradshaw LE, Kearney FC, Russell C, Whittamore KH, Foster PE, Mamza J, Gladman JR, Jones RG, Lewis SA, Porock D, Harwood RH (2013). Care in specialist medical and mental health unit compared with standard care for older people with cognitive impairment admitted to general hospital: randomised controlled trial (NIHR TEAM trial). BMJ.

[22] Briggs R, Coary R, Collins R, Coughlan T, O’Neill D, Kennelly SP (2016). Acute hospital care: how much activity is attributable to caring for patients with dementia?. QJM.

[23] Kim SYH (2011). The ethics of informed consent in Alzheimer disease research. Nat Rev Neurol.

[24] Alzheimer Europe (2011) Informed consent to dementia research. http://www.alzheimer-europe.org/Ethics/Ethical-issues-in-practice/2011-Ethics-of-dementia-research/Informed-consent-to-dementia-research. Accessed 4 Jan 2017

[25] Kenward MG (2013). The handling of missing data in clinical trials. Clin Investig.

[26] Chan A-W, Altman DG (2005). Identifying outcome reporting bias in randomised trials on PubMed: review of publications and survey of authors. BMJ.

[27] Trevillion K, Oram S, Feder G, Howard LM (2012). Experiences of domestic violence and mental disorders: a systematic review and meta-analysis. PLoS One.

[28] Dwan K, Altman DG, Arnaiz JA, Bloom J, Chan A-W, Cronin E, Decullier E, Easterbrook PJ, Von Elm E, Gamble C, Ghersi D, Ioannidis JPA, Simes J, Williamson PR (2008). Systematic review of the empirical evidence of study publication bias and outcome reporting bias. PLoS One.

[29] National Health Service Improvement (2017) Dementia assessment and improvement framework. Available from https://improvement.nhs.uk/resources/dementia-assessment-and-improvement-framework/. Accessed Dec 2017

